# Excitatory subtypes of the lateral amygdala neurons are differentially involved in regulation of synaptic plasticity and excitation/inhibition balance in aversive learning in mice

**DOI:** 10.3389/fncel.2023.1292822

**Published:** 2023-12-14

**Authors:** Mieko Morishima, Sohta Matsumura, Suguru Tohyama, Takashi Nagashima, Ayumu Konno, Hirokazu Hirai, Ayako M. Watabe

**Affiliations:** ^1^Institute of Clinical Medicine and Research, Research Center for Medical Sciences, The Jikei University School of Medicine, Chiba, Japan; ^2^Gunma University Graduate School of Medicine, Maebashi, Japan; ^3^Viral Vector Core, Gunma University Initiative for Advanced Research (GIAR), Maebashi, Japan

**Keywords:** lateral amygdala (LA), medial geniculate nucleus (MGN), excitatory cell, inhibitory cell, fear conditioning, experience-dependent plasticity, inhibition/excitation

## Abstract

The amygdala plays a crucial role in aversive learning. In Pavlovian fear conditioning, sensory information about an emotionally neutral conditioned stimulus (CS) and an innately aversive unconditioned stimulus is associated with the lateral amygdala (LA), and the CS acquires the ability to elicit conditioned responses. Aversive learning induces synaptic plasticity in LA excitatory neurons from CS pathways, such as the medial geniculate nucleus (MGN) of the thalamus. Although LA excitatory cells have traditionally been classified based on their firing patterns, the relationship between the subtypes and functional properties remains largely unknown. In this study, we classified excitatory cells into two subtypes based on whether the after-depolarized potential (ADP) amplitude is expressed in non-ADP cells and ADP cells. Their electrophysiological properties were significantly different. We examined subtype-specific synaptic plasticity in the MGN-LA pathway following aversive learning using optogenetics and found significant experience-dependent plasticity in feed-forward inhibitory responses in fear-conditioned mice compared with control mice. Following aversive learning, the inhibition/excitation (I/E) balance in ADP cells drastically changed, whereas that in non-ADP cells tended to change in the reverse direction. These results suggest that the two LA subtypes are differentially regulated in relation to synaptic plasticity and I/E balance during aversive learning.

## Introduction

Aversive learning is essential for animal survival. In associative learning, such as Pavlovian fear conditioning, an emotionally neutral conditioned stimulus (CS), such as an auditory tone, acquires the ability to elicit defensive behaviors after being associated with an innately aversive unconditioned stimulus (US), such as a foot shock (LeDoux, 2000; Maren and Quirk, 2004). Convergence of the CS and US occurs in the lateral amygdala (LA), a brain region crucial for fear memory formation (Davis, 1992; Li et al., 1996; Fanselow and LeDoux, 1999; LeDoux, 2000; Maren, 2000). Long-term potentiation (LTP) has long been postulated as a circuit model for some forms of associative learning (Bliss and Collingridge, 1993). Accumulating evidence has shown that LTP occurs in pathways that transmit CS information to the LA, such as the pathway from the medial geniculate nucleus (MGN) of the thalamus to the LA, during fear memory formation (McKernan and Shinnick-Gallagher, 1997; Kwon et al., 2014; Nabavi et al., 2014). Furthermore, blockade of the extracellular signal-regulated kinase/mitogen-activated protein kinase signaling cascade, a key component in LTP regulation, also disrupts aversive learning in the LA (Schafe et al., 2000; Johansen et al., 2011). This line of evidence suggests that associative plasticity in the LA may be the underlying mechanism of the CS-US association in Pavlovian aversive learning (Rogan et al., 1997).

Excitatory cells are the major group of neurons in the LA. It is well known that excitatory cells are heterogeneous in their firing patterns, projection patterns, morphologies, and transcriptomes (Larkman and Mason, 1990; DeFelipe and Farinas, 1992; Morishima and Kawaguchi, 2006; Otsuka and Kawaguchi, 2008; McGarry and Carter, 2017; O’Leary et al., 2020; Yao et al., 2021). Inhibitory cells, on the other hand, are minor groups of neurons that are rich in cell types and are classified by their firing patterns, chemical markers, morphologies, and transcriptomes (Kawaguchi and Kubota, 1997; Markram et al., 2004; Sosulina et al., 2010; Polepalli et al., 2020; Scala et al., 2021; Vereczki et al., 2021; Yu et al., 2023). These heterogeneities contribute to differential functional regulation and connectivity patterns. Excitatory neurons in the LA are traditionally classified based on their firing patterns (Faber et al., 2001; Sosulina et al., 2006). However, there are few reports on the relationship between excitatory subtypes and other functional properties of the LA.

In many brain regions, including the sensory cortex and the hippocampus, excitatory and feed-forward or feed-back inhibitory synaptic inputs into excitatory cells are precisely controlled. While the excitation/inhibition balance is fairly constant in the basal state, it can be modulated by experience-dependent plasticity (Maffei et al., 2004; House et al., 2011; Isaacson and Scanziani, 2011). The excitation/inhibition balance plays an important role in shaping the temporal window for spike generation and activity gating (Buzsaki, 1984; Wehr and Zador, 2003; Bartley et al., 2015). Although the regulation of excitation/inhibition balance has been investigated in various brain regions and cell types, how synaptic plasticity in aversive learning affects the regulation of the excitation/inhibition balance in a subtype-specific manner remains to be elucidated.

In this study, we classified the excitatory cells into two subtypes based on their ADP amplitude, which differs depending on the firing patterns (Connors and Gutnick, 1990; Graves et al., 2012): non-ADP cells and ADP cells. The electrophysiological properties of the two subtypes differed significantly. We hypothesized that aversive learning affects the regulation of excitatory-inhibitory balance in a subtype-specific manner. To address this issue directly, we performed *in vitro* whole-cell patch-clamp recordings from fear-conditioned and control mice. The present results suggest that the two LA subtypes are differentially regulated in relation to synaptic plasticity and the I/E balance following aversive learning.

## Materials and methods

### Animals

All the experimental protocols in this study, including animal use, were approved by the Institutional Animal Care and Use Committee of Jikei University (Tokyo, Japan; Approval No. 2019-045). All experiments complied with the Guidelines for Proper Conduct of Animal Experiments by the Science Council of Japan (2006) and recommendations of the International Association for the Study of Pain. All efforts were made to reduce the number of animals used and the suffering of the animals. Mice were group-housed and maintained on a 12-h light/dark cycle with food and water *ad libitum*. Male C57BL/6J mice (Japan SLC, Shizuoka, Japan) were used for the slice recording and behavior test. Both male and female Ai14 mice (Jax: Stock No:13044) were used for labeling GAD-positive cells and slice recording. The number of Ai14 mice was limited; therefore, we compared the ratio of inhibitory neuronal subtypes in male and female Ai14 mice. Ai14 male; RS: *n* = 13, 48.4%, Burst: *n* = 9, 32.3%, Late: *n* = 3, 9.7%, AC: *n* = 3, 9.7% from *N* = 7 mice. Ai14 female; RS: *n* = 13, 47.8%, Burst: *n* = 8, 30.4%, Late: *n* = 2, 8.7%, AC: *n* = 3, 13.0% from *N* = 5 mice. Because they were of similar proportions, we included both male and female data.

### Viral injection

AAV1-hSyn-Chronos-GFP (4.6 × 10^12^ vg/mL, Klapoetke et al., 2014) was purchased from UNC Vector Core. AAV1-mGAD65-Cre (8.9 × 10^12^ vg/mL, Hoshino et al., 2021) was supplied by Viral Vector Core, Gunma University Initiative for Advanced Research (GIAR). Mice (4–5 weeks old) were intraperitoneally anesthetized with a mixture of 0.75 mg/kg medetomidine hydrochloride (Zenoaq, Fukushima, Japan), 4.0 mg/kg midazolam (Astellas, Tokyo, Japan), and butorphanol tartrate (5.0 mg/kg; Meiji Seika Pharma, Tokyo, Japan) and fixed in a stereotaxic device (Narishige, Tokyo, Japan). AAV1-hSyn-Chronos-GFP was bilaterally microinjected into the medial geniculate nucleus (MGN) from bregma: AP –2.9; ML; 1.8–1.9; DV –2.9 mm of C57BL/6J mice using a Hamilton microsyringe (1701RN Neuros Syringe, 33 G, 10 μl; Hamilton Company, Reno, NV, USA). AAV1-mGAD65-Cre were injected into Ai14 mice in the same manner as above. The injection and axonal projection sites were observed using an FV3000 confocal microscope (Olympus, Tokyo, Japan) with 488 nm excitation laser light or a fluorescence microscope (BZ-X700, Keyence, Osaka, Japan).

### Slice preparation

Mice (8–10 weeks old) were deeply anesthetized with isoflurane (5%) and transcardially perfused with ice-cold oxygenated (95% O_2_ and 5% CO_2_) cutting solution before decapitation. Coronal brain slices (300 μm), including the LA, were cut with a vibratome (VT1200S, Leica, Wetzlar, Germany) in an ice-cold cutting solution containing (in mM) 2.5 KCl, 0.5 CaCl_2_, 10 MgSO4, 1.25 NaH_2_PO_4_, 3 sodium pyruvate, 92 N-methyl-D-glucamine, 20 HEPES, 12 N-acetyl-L-cysteine, 25 D-glucose, 5 L-ascorbic acid, and 30 NaHCO_3_ equilibrated with 95% O_2_ plus 5% CO_2_. The slices were recovered in the same cutting solution at 34°C after 10–15 min. The slices were transferred to the 20–25°C standard artificial cerebrospinal fluid (ACSF) containing (in mM): 125 NaCl, 3 KCl, 2 CaCl_2_, 1.3 MgCl_2_, 1.25 NaH_2_PO_4_, 10 D-glucose, 0.4 L-ascorbic acid, and 25 NaHCO3 (bubbled with 95% O_2_ + 5% CO_2_) and kept until recording.

### Electrophysiology

Whole-cell patch-clamp recordings were performed in LA neurons, which were visually identified under an upright microscope with oblique illumination (BX-51WI, Olympus). Patch-clamp electrodes (4–8 MΩ) were made of borosilicate glass pipettes (1B150F-4, World Precision Instruments, Sarasota, FL, USA). The internal solution contained (in mM) 120 potassium gluconate, 10 HEPES, 7 KCl, 0.2 EGTA, 4 NaCl, 2 MgATP, and 0.3 NaGTP and 0.4% Biocytin (pH 7.2 as adjusted with KOH; osmolarity, 295–300 mOsm/kg). Light-evoked (le) excitatory postsynaptic currents (EPSCs) were recorded at a holding potential of –60 mV in the ACSF. Because of the necessity of measuring the ADP amplitude, we used a K-based internal solution and recorded IPSCs at −40 mV. We did not observe any unclamped current when recording with voltage-clamp at −40 mV. Chronos was activated using an LED (470 nm) illumination system (470L4, Thorlabs, Newton, NJ, USA) through a 40 × water-immersion objective lens (LUMPLFLN40XW, NA 0.8; Olympus). LED stimulation was applied for 5 ms every 20 s. Photo-stimulation was controlled using Master 8 (A.M.P.I., Jerusalem, Israel). The paired-pulse ratio was defined as the ratio of the second EPSC amplitude to the first EPSC amplitude in response to two stimuli with a 100 ms interstimulus interval. The data analyses were performed with blinding of the experimental group.

The properties of after-depolarization (ADP), resting membrane potential (rmp), threshold, half-width of action potential (AP), AP amplitude, after-hyperpolarized potential amplitude (AHP), AHP time, Ri, and sag ratio, and initial inter-spike interval (i-ISI) were measured with the current-clamp in the ACSF. To measure AP properties, we injected 10 pA step-depolarizing currents (duration 1 s, amplitude 10–100 pA). We used the AP from a step-depolarizing current that allowed single AP generation. Rmp was measured at 200 ms before the current injection. Ri was obtained by linear fitting of voltage changes from 0 to −30 pA pulses at 10 pA steps. A threshold was a voltage of AP onset. AP amplitude was the difference between the threshold and peak of AP voltage. AP half-width was the time between half of an AP amplitude. The AHP was defined as the difference between the threshold and the most negative voltage observed within a 5 ms window. AHP time was the duration from reaching the threshold to the AHP. The fast component of the AHP (fAHP) is referred to as the fast negative peak. ADP was the difference in amplitude between the peak occurring between fAHP and AHP, and the threshold voltage. We defined ADP neurons as having an amplitude of ADP larger than three times the calculated SD of noise (0.703 mV, *n* = 30 cells from *N* = 30 mice) to eliminate noise contamination. The sag ratio was determined by comparing the differences between the peak and steady state induced by hyperpolarizing currents (1 s duration, −50 pA), divided by the peak amplitude. I-ISI represented the time interval between the first and second spikes of the firing in response to depolarizing currents (1 s duration, 200 pA). The data were discarded when the series resistance of the recorded cells was higher than 35 MΩ. To classify the inhibitory cells, we used the parameters: rmp, threshold, AP amplitude, half-width of AP, AHP, AHP time, Ri, i-ISI, and sag ratio and performed cluster analysis using Ward’s method (Ward, 1963) by Python scikit-learn. The membrane currents and membrane potentials were recorded with a Sutter Patch Amplifier (Sutter Instrument, Novato, CA, USA), filtered at 5 kHz. The data were analyzed by Igor (Wave Metrics, Portland, OR, USA) and Sutter Patch software (Sutter Instrument). The I/E ratio was calculated as the le-IPSCs divided by the le-EPSCs amplitude recorded in the same neuron.

### Morphological analysis

After recording, the slices were fixed with 4% paraformaldehyde in 0.01 M phosphate-buffered saline (PBS) solution overnight. Slices were then washed in 0.01 M PBS three times. After washing the slices, we stained the recorded cells with streptavidin combined with the Scale methods (Hama et al., 2015). For clarification, we used SCALEVIEW^®^-S (Fujifilm Wako, Osaka, Japan). Briefly, the slices were incubated in the ScaleView-S0, S1, and S2 for 20 min, 8 M urea for 2 h, and ScaleView-S2 for 30 min at 37°C. After being washed in PBS for 30 min, the slices were incubated in 1:500 streptavidin Alexa Fluor 594 (S11227, Thermo Fisher Scientific, Waltham, MA, USA) or 647 (S32357, Thermo Fisher Scientific) in AB Scale solution overnight. The slices were washed with AB Scale solution for 30 min, fixed with 4% paraformaldehyde for 5 min, and washed with 0.01 M PBS, and then, the slices were mounted in ScaleView-S4.

Fluorescent images of the stained cells were acquired using a confocal microscope (FV3000, Olympus) with 594- or 647-nm-excitation laser light. The cells were reconstructed three-dimensionally using the Neurolucida workstation system (MicroBrightField, Williston, VT, USA). The reconstructed neurons were analyzed using NeuroExplorer (MicroBrightField).

### Fear conditioning

Male mice (8–10 weeks old) were habituated to handling for more than 3 days before fear conditioning. All the conditioning procedures were conducted in a conditioning chamber (170 mm width × 100 mm depth × 100 mm height, with transparent acrylic wall and floor metal grids; 200 Lux, 60 dB background white noise) surrounded by a sound-attenuating chamber (CL-M3, O’Hara & Co., Ltd., Tokyo, Japan). To be habituated to tone CS, the animals were placed in a conditioning chamber and received six presentations of CS (4 kHz, 65 dB, 30 s) 1 day before the conditioning session. Following a 270 s acclimation period, the mice received three presentations of tone CS (4 kHz, 65 dB, 30 s), each co-terminating with a foot shock US (0.6 mA, 2 s) with a randomized inter-stimulus interval (100–140 s) presented in a conditioning chamber. Con mice received only the tone. A retrieval test was conducted 24 h later in a retrieval chamber (170 mm width × 100 mm depth × 100 mm height, with white acrylic plate walls scented with peppermint odor and sandpaper on the floor; 200 Lux, 50 dB background white noise). Animals received CS for 30 s six times in a retrieval chamber. The CS presentation began 270 s after the mice were placed in the chamber.

### Statistic analysis

All data were analyzed using GraphPad Prism 9 software (GraphPad Software, Boston, MA, USA). For each figure, data are presented as means ± SEM. Paired data were compared using paired *t*-tests. Unpaired data were tested using the Mann–Whitney U test. Unmatched multiple data were tested using the Kruskal–Wallis test with Dunn’s *post-hoc* test. Two-way ANOVA (with *post-hoc* Sidak’s test) was used for the fear conditioning analysis. Two-way ANOVA (with *post-hoc* Bonferroni’s test) was used for the Sholl analysis. Correlations were analyzed using Pearson’s (r). Significance was accepted at *p* < 0.05. *n* = cell number and *N* = animal number.

## Results

### Two distinct excitatory subtypes in the LA

**FIGURE 1:**
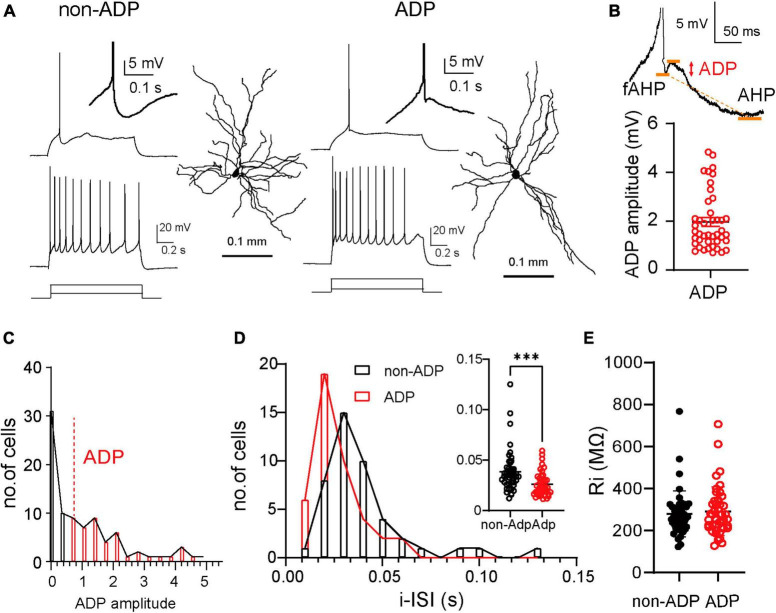
Two distinct firing subtypes in the lateral amygdala. **(A)** Example of a single action potential (AP) and repetitive firing patterns to depolarized current injection (non-ADP cells: 80 pA and 200 pA; ADP cells: 50 pA and 200 pA) and dendritic morphologies of the excitatory non-ADP and ADP cells. Inset showed non-ADP and ADP. **(B)** ADP measured the peak between the fast AHP (fAHP) and AHP. The plots showed the ADP amplitude (*n* = 43 cells from *N* = 26 mice). **(C)** The distribution of the ADP in all cells (non-ADP cells: *n* = 44 cells from *N* = 24 mice; ADP cells: *n* = 43 cells from *N* = 26 mice). **(D)** The histogram of the initial inter-spike interval (i-ISI) of the non-ADP (black) and ADP cells (red). Inset showed the comparison of the ADP between the excitatory subtypes (non-ADP cells: *n* = 44 cells from *N* = 24 mice; ADP cells: *n* = 43 cells from *N* = 26 mice; *p* = 0.0003). **(E)** The Ri of the non-ADP cells was similar to that of ADP cells (non-ADP cells: *n* = 44 cells from *N* = 24 mice; ADP cells: *n* = 43 cells from *N* = 26 mice; *p* = 0.60). ***p < 0.001.

The LA consists of more than 85% excitatory cells and less than 15% inhibitory cells (Pare et al., 1995; Polepalli et al., 2020; Vereczki et al., 2021) that can be identified by their firing patterns. Previous reports in rats have shown that excitatory cells are classified according to their firing patterns into adaptation and non-adaptation types based on spike frequency adaptation in response to current injection (Faber et al., 2001; Sosulina et al., 2006). Inhibitory cells exhibit very different firing patterns; for example, they have a larger AHP amplitude (Polepalli et al., 2020) and a higher firing frequency (Vereczki et al., 2021). We occasionally patched inhibitory cells (20 inhibitory cells out of a total of 107 cells from *N* = 14 mice) and discarded them based on these criteria. The afterdepolarization (ADP) of action potentials (AP) in excitatory cells differs depending on the firing patterns and regulates excitability (Connors and Gutnick, 1990; Metz et al., 2005; Higgs and Spain, 2009; Graves et al., 2012). Thus, we examined whether ADP could be used as a classification parameter for LA excitatory cells. We performed whole-cell patch-clamp recordings using a biocytin-filled internal solution from acute LA slices obtained from C57BL/6J mice. The excitatory cells exhibited different firing patterns depending on the ADP amplitude (Figure 1A). We divided the excitatory cells into two groups, non-ADP and ADP cells, based on whether they had an ADP (above 0.7 mV). The mean amplitude of ADP was 1.97 ± 0.018 mV (*n* = 43 cells from *N* = 26 mice; Figure 1B). They distributed from 0.716 mV to 4.8 mV (black bar: non-ADP: *n* = 44 cells from *N* = 24 mice, red bar: ADP: *n* = 43 cells from *N* = 26 mice; Figure 1C). We next investigated the initial inter-spike interval (i-ISI) because it has been reported that the i-ISI of the firing pattern differs depending on the amplitude of ADP (Graves et al., 2012). Then, we found that the ADP cells had shorter i-ISI than the non-ADP (non-ADP: 0.039 ± 0.021 s, *n* = 44 cells from *N* = 24 mice, ADP: 0.026 ± 0.012 s, *n* = 43 cells from *N* = 26 mice, Mann–Whitney U test, *p* = 0.0003; Figure 1D). The Ri did not differ between the two excitatory subtypes (non-ADP: 278.7 ± 16.43 MΩ, *n* = 44 cells from *N* = 24 mice, ADP: 291.6 ± 17.78 MΩ, *n* = 43 cells from *N* = 26 mice, Mann–Whitney U test, *p* = 0.60; Figure 1E).

### Different intrinsic properties between the non-ADP and ADP cells

**FIGURE 2:**
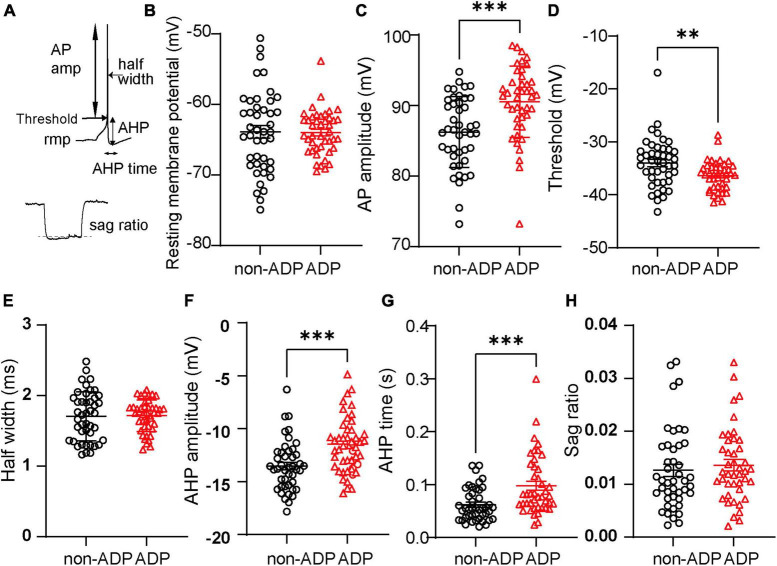
Different intrinsic properties of the two types of RS cells. **(A)** Seven physiological parameters used for the analysis were resting membrane potential (rmp), AP amplitude, threshold, half-width of action potential (AP), after-hyperpolarized potential amplitude (AHP), AHP time, and sag ratio. **(B)** The resting membrane potentials (rmp) were similar between the two excitatory subtypes (non-ADP cells: *n* = 44 cells from *N* = 24 mice; ADP cells: *n* = 43 cells from *N* = 26 mice; *p* = 0.87). **(C)** The AP amplitude of ADP cells was larger than that of the non-ADP cells (non-ADP cells: *n* = 44 cells from *N* = 24 mice; ADP cells: *n* = 43 cells from *N* = 26 mice; *p* = 0.0001). **(D)** Threshold of action potential (AP) in the ADP cells was significantly deeper than in the non-ADP cells (non-ADP cells: *n* = 44 cells from *N* = 24 mice; ADP cells: *n* = 43 cells from *N* = 26 mice; *p* = 0.0023). **(E)** The half-width of the AP in the non-ADP cells was similar to that in the ADP cells (non-ADP cells: *n* = 44 cells from *N* = 24 mice; ADP cells: *n* = 43 cells from *N* = 26 mice; *p* = 0.82). **(F)** The AHP amplitudes significantly differed between the two excitatory subtypes (non-ADP cells: *n* = 44 cells from *N* = 24 mice; ADP cells: *n* = 43 cells from *N* = 26 mice; *p* = 0.0002). **(G)** The AHP time of non-ADP cells were significantly faster than that of ADP cells (non-ADP cells: *n* = 44 cells from *N* = 24 mice; ADP cells: *n* = 43 cells from *N* = 26 mice; *p* = 0.0003). **(H)** The sag ratio was similar between the two excitatory subtypes (non-ADP cells: *n* = 44 cells from *N* = 24 mice; ADP cells: *n* = 43 cells from *N* = 26 mice; *p* = 0.34). **p < 0.01, ***p < 0.001.

To further characterize the physiological properties of non-ADP and ADP cells, we examined the intrinsic properties of a single AP and sag ratio in response to the current injection of the recorded cells (Figure 2A, Materials and Methods). non-ADP cells had similar rmp with ADP cells; the mean rmp of non-ADP cells was −63.89 ± 0.88 mV, and that of ADP cells was −64.0 ± 0.48 mV (non-ADP cells: *n* = 44 cells from *N* = 24 mice, ADP cells: *n* = 43 cells from *N* = 26 mice, Mann–Whitney U test, *p* = 0.87; Figure 2B). The AP amplitude of ADP cells was larger than that of non-ADP (non-ADP: 86.22 ± 0.76 mV, *n* = 44 cells from *N* = 24 mice, ADP: 90.52 ± 0.77 mV, *n* = 43 cells from *N* = 26 mice, Mann–Whitney U test, *p* = 0.0001; Figure 2C). The threshold of ADP cells was significantly more negative than that of non-ADP cells (non-ADP: −34.0 ± 0.69 mV, *n* = 44 cells from *N* = 24 mice, ADP: −36.41 ± 0.42 mV, *n* = 43 cells from *N* = 26 mice, Mann–Whitney U test, *p* = 0.0023; Figure 2D). On the other hand, the AP half-width of non-ADP cells (1.71 ± 0.052 ms, *n* = 44 cells from *N* = 24 mice) was similar to that of ADP cells (1.72 ± 0.034 ms, *n* = 43 cells from *N* = 26 mice, Mann–Whitney U test, *p* = 0.82; Figure 2E). Previous reports have shown that the firing classification of excitatory cells based on their spike frequency and adaptation could account for AHP amplitude in rats (Faber et al., 2001; Faber and Sah, 2002). Our classification based on the ADP amplitude also showed significant differences in AHP amplitude between non-ADP and ADP cells in mice (non-ADP: –13.51 ± 0.36 mV, *n* = 44 cells from *N* = 24 mice, ADP: −11.45 ± 0.40 mV, *n* = 43 cells from *N* = 26 mice, Mann–Whitney U test, *p* = 0.0002; Figure 2F). The time from threshold to AHP (AHP time) of ADP cells were shorter than that of non-ADP (non-ADP: 0.062 ± 0.005 s, *n* = 44 cells from *N* = 24 mice, ADP: 0.098 ± 0.009 s, *n* = 43 from *N* = 26 mice, Mann–Whitney U test, *p* = 0.0003; Figure 2G). There was no significant difference in the sag ratio between the two excitatory cells (non-ADP: 0.013 ± 0.008 s, *n* = 44 cells from *N* = 24 mice, ADP: 0.014 ± 0.007, *n* = 43 from *N* = 26 mice, Mann–Whitney U test, *p* = 0.34; Figure 2H). Thus, we revealed that ADP cells had more negative threshold and smaller AHP and larger AP amplitude than non-ADP cells.

### Similar morphological properties of the two subtypes

**FIGURE 3:**
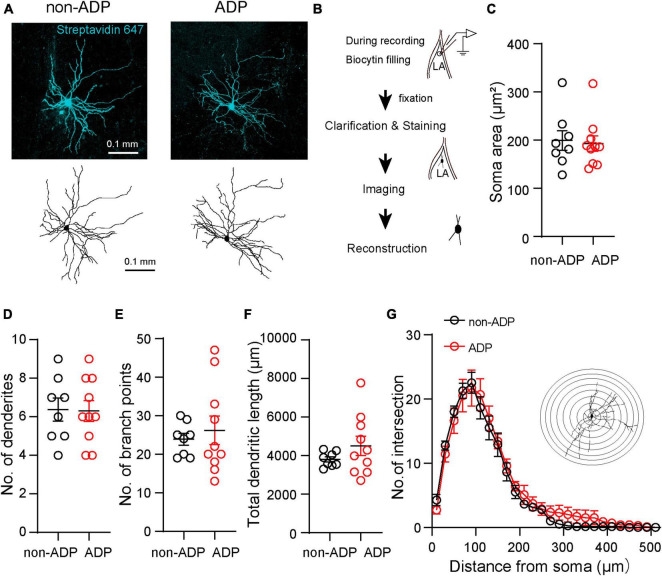
Morphological features of the two excitatory subtypes. **(A)** Confocal images and reconstructions of the dendrites and soma of the non-ADP (left) and ADP (right) cells. **(B)** Scheme of the morphological analysis. **(C)** The soma area of the non-ADP cells was similar to that of the ADP cells (black circle: non-ADP cells, *n* = 8 from *N* = 5 mice; red circle: ADP cells, *n* = 10 from *N* = 7 mice; *p* = 0.83). **(D)** The number of dendrites was similar between the non-ADP and ADP cells (black circle: non-ADP cells: *n* = 8 from *N* = 5 mice; A red circle: DP cells: *n* = 10 from *N* = 7 mice; *p* = 0.97). **(E)** The non-ADP cells had similar branch points of dendrites with the ADP cells (n black circle: on-ADP cells: *n* = 8 from *N* = 5 mice; red circle: ADP cells: *n* = 10 from *N* = 7 mice; *p* = 0.92). **(F)** The non-ADP cells had a similar dendritic length to the ADP cells (black circle: non-ADP cells: *n* = 8 from *N* = 5 mice; A red circle: DP cells: *n* = 10 from *N* = 7 mice; *p* = 0.51). **(G)** Sholl analysis of the number of intersections of non-ADP and ADP cells (Two-Way ANOVA with *post-hoc* Bonferroni test, all bins: *p* > 0.99). The inset shows an illustration of Sholl analysis. Concentric circles started 10 μm from the center of the soma and increased in radius by 20 μm.

Next, we examined whether electrophysiologically distinct excitatory subtypes, non-ADP and ADP cells, had different morphological properties. To quantitatively analyze the morphological properties of the excitatory subtypes, we used an intracellular solution containing biocytin during whole-cell patch-clamp recordings and staining with streptavidin. After staining the slices, combined with the Scale methods (Hama et al., 2015) and imaging of the recorded cells, we reconstructed their morphology using Neurolucida (Figures 3A, B). We confirmed that the stained cells contained spines in their dendrites. First, we compared their soma size, and there were no significant differences between them (non-ADP: 199.4 ± 20.48 μm^2^, *n* = 8 from *N* = 5 mice, ADP: 192.9 ± 16.03 μm^2^, *n* = 10 from *N* = 7 mice, Mann–Whitney U test, *p* = 0.83; Figure 3C). The number of dendrites was similar in both subtypes (non-ADP: no. = 6.38 ± 0.60, *n* = 8 from *N* = 5 mice, ADP: no. = 6.30 ± 0.54, *n* = 10 from *N* = 7 mice, Mann–Whitney U test, *p* = 0.97; Figure 3D). Next, when the number of branching points, a measure of complexity, was examined, no significant difference was found between the two excitatory subtypes (non-ADP: no. = 23.88 ± 1.52, *n* = 8 from *N* = 5 mice, ADP: no. = 26.2 ± 3.72, *n* = 10 from *N* = 7 mice, Mann–Whitney U test, *p* = 0.92; Figure 3E). The total dendritic length of the non-ADP cells was also similar with the ADP cells (non-ADP: 3.81 ± 1.35 mm, *n* = 8 from *N* = 5 mice, ADP: 4.51 ± 0.50 mm, *n* = 10 from *N* = 7 mice, Mann–Whitney U test, *p* = 0.51; Figure 3F). We also analyzed the complexity of the dendrites using Sholl analysis. There were no statistically significant differences in the number of intersections (Two-way ANOVA with Bonferroni’s *post-hoc* test, *p* > 0.999; Figure 3G). These results showed that the non-ADP cells and the ADP cells were not significantly different in their morphology.

### Fear conditioning did not significantly affect the synaptic input patterns from the MGN

**FIGURE 4:**
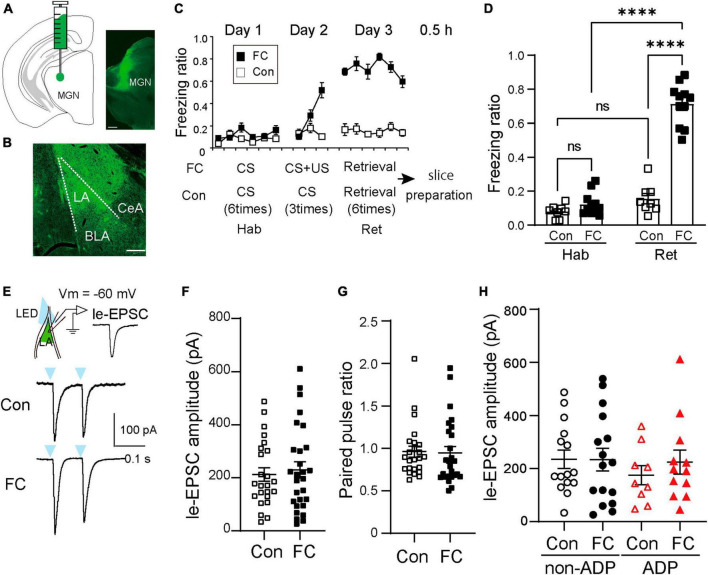
The auditory fear conditioning unalters the thalamic-driven excitation of both excitatory subtypes. **(A)** Schematic of AAV1-hSyn-Chronos-GFP injection into the medial geniculate nucleus (MGN). Scale bar: 0.5 mm. **(B)** Images of the axonal termination of the AAV1-hSyn-Chronos-GFP in the LA. Scale bar: 0.2 mm. **(C)** Experimental diagram of sound-cued fear conditioning. The mice were habituated to tone the CS on day 1 (Hab). Following the 270 s acclimation period, FC mice (black filled square: *N* = 11) received three presentations of tone, CS, each co-terminating with a foot shock, US, Con mice (square: *N* = 8) received only the tone on day 2. Acute LA slices were prepared at 0.5 h after a retrieval test (Ret). **(D)** The freezing ratio in the retrieval test was higher than in the habituation test (*N* = 11; *p* < 0.0001). During the retrieval test, the freezing ratio of the FC mice was significantly higher than that of the Con mice (*N* = 8). **(E)** Light-evoked EPSCs (le-EPSCs) are evoked by LED stimulation (every 20 s, 5 ms duration) at –60 mV. **(F)** The le-EPSCs of all RS cells in FC mice (*n* = 28 from *N* = 11 mice) were similar to those in Con mice (*n* = 24 from *N* = 8 mice; *p* = 0.86). **(G)** Paired-pulse ratio: The two successive le-EPSCs ratios (inter-pulse interval; 100 ms) of both types of RS cells did not differ between Con (*n* = 24 from *N* = 8 mice) and FC mice (*n* = 28 from *N* = 11 mice, *p* = 0.42). **(H)** Comparison of the le-EPSCs amplitudes in Con and FC mice within the same subtypes and comparison of the le-EPSCs in Con or FC mice in the different subtypes. There were no significant differences (Con mice, non-ADP: *n* = 15 from *N* = 6 mice, ADP: *n* = 9 from *N* = 7 mice, Kruskal–Wallis test with Dunn’s *post-hoc* test, *p* > 0.99, FC mice, non-ADP: *n* = 16 from *N* = 9 mice, ADP: *n* = 12 from *N* = 7 mice, Kruskal–Wallis test with Dunn’s *post-hoc* test, *p* > 0.99). ****p < 0.0001.

Auditory fear conditioning can induce synaptic plasticity in the LA excitatory neurons. However, little is known about subtype-dependent regulation of plasticity in the MGN-LA pathway. To examine whether the two excitatory subtypes described above were differentially regulated in aversive learning, we injected AAV1-hSyn-Chronos-GFP into the MGN of mice (Figure 4A) for analyzing plasticity in the MGN-LA pathway. Four to five weeks after AAV injection, Chronos-GFP fluorescence was observed in the LA (Figure 4B; Doron and Ledoux, 1999; Kwon et al., 2014). We then conducted fear conditioning on AAV1-hSyn-Chronos-GFP-injected mice (Figure 4C). Fear-conditioned mice (FC mice) displayed auditory tone-evoked freezing (Two-way ANOVA with Sidak *post-hoc* test, *p* < 0.001; Figures 4C, D). Within 0.5 h, we prepared acute slices and performed whole-cell recordings from the FC mice (*N* = 11). As a control, AAV1-hSyn-Chronos-GFP-injected control mice (Con: *N* = 8, Figure 4C) were used. We next tested whether aversive learning affected the excitatory inputs from the MGN into the two excitatory subtypes. Previous studies have reported aversive learning induced synaptic plasticity in the pathway from the MGN to the LA (Goosens et al., 2003). After fear conditioning, rat LA neurons *in vitro* show larger postsynaptic excitatory currents in response to MGN-LA pathway stimulation than the control mice or rats (McKernan and Shinnick-Gallagher, 1997; Clem and Huganir, 2010; Kim and Cho, 2017; Jeong et al., 2021). However, it remains unclear whether aversive learning leads to changes in the synaptic properties of thalamic inputs depending on the two excitatory subtypes. To examine this question, we first compared the le-EPSCs amplitude (Figure 4E) between the Chronos-GFP-injected Con and FC mice, which specifically represented inputs from the MGN-LA pathway (Figure 4B). Notably, the le-EPSCs amplitude in FC mice closely resembled Con mice (Con: 212.20 ± 25.60 pA, *n* = 24 from *N* = 8 mice, FC: 229.20 ± 30.60 pA, *n* = 28 from *N* = 11 mice, Mann–Whitney U test, *p* = 0.86; Figure 4F). To examine the change in presynaptic release probability, we recorded two successive le-EPSCs (inter-pulse interval; 100 ms). The paired-pulse ratio in Con mice did not significantly differ from that in FC mice (Con: 0.96 ± 0.065, *n* = 24 from *N* = 8 mice, FC: 0.94 ± 0.076, *n* = 28 from *N* = 11 mice, Mann–Whitney U test, *p* = 0.31; Figures 4E, G). To examine the possibility that MGN synaptic weights were different in a target-dependent manner, we compared the le-EPSCs amplitudes between the non-ADP and ADP cells within the Con mice. There were no significant differences between the non-ADP cells and the ADP cells in the Con mice (non-ADP: 234.40 ± 34.40 pA, *n* = 15 from *N* = 6 mice, ADP: 174.70 ± 35.95 pA, *n* = 9 from *N* = 7 mice; Kruskal–Wallis test with Dunn’s *post-hoc* test, *p* > 0.99; Figure 4H). Similarly, within the FC mice the le-EPSCs amplitudes of the non-ADP cells closely resembled those of the ADP cells (non-ADP: 233.40 ± 42.72 pA, *n* = 16 from *N* = 9 mice; ADP: 223.60 ± 45.18 pA, *n* = 12 from *N* = 7 mice; Kruskal–Wallis test with Dunn’s *post-hoc* test, *p* > 0.99; Figure 4H). In both excitatory subtypes, the le-EPSC amplitudes in the FC mice exhibited similarities to those in the Con mice (non-ADP, Kruskal–Wallis test with Dunn’s *post-hoc* test, *p* > 0.99; ADP, Kruskal–Wallis test with Dunn’s *post-hoc* test, *p* > 0.99; Figure 4H). These results suggest that thalamic inputs to excitatory subtypes are subject to similar regulatory mechanisms.

### Fear conditioning induces E/I balance shifts in an excitatory subtype-specific manner

**FIGURE 5:**
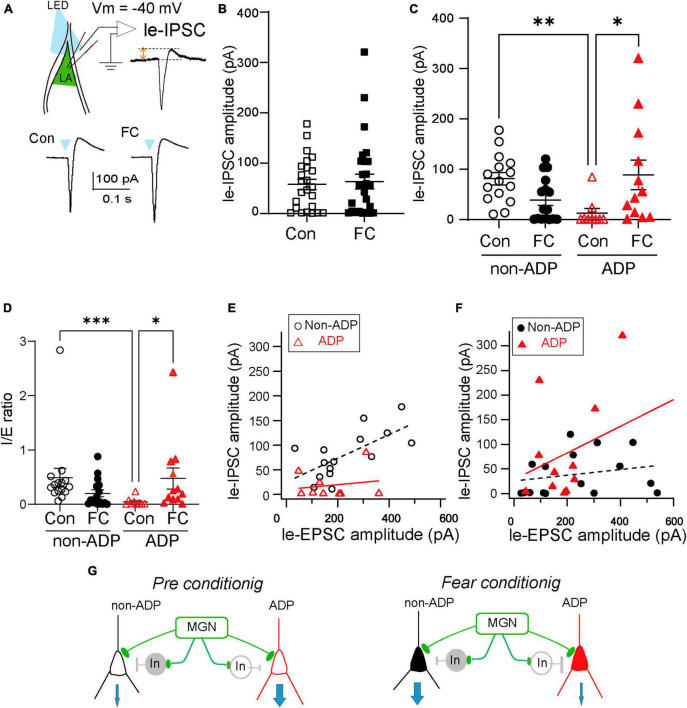
The E/I balance variation depended on the excitatory subtypes. **(A)** The illumination of LED (every 20 s, 5 ms duration) evoked le-EPSCs followed by le-IPSCs in the excitatory cells at holding potentials –40 mV. **(B)** The le-IPSCs amplitude of all RS cells in FC mice (*n* = 28 from *N* = 11 mice) was similar to those in Con mice (*n* = 24 from *N* = 8 mice; *p* = 0.79). **(C)** Comparison of the le-IPSCs amplitude in Con and FC mice within the same subtypes and comparison of the subtype differences in the le-IPSCs amplitude from Con and FC mice. The amplitude of le-IPSCs of ADP cells in the FC mice (*n* = 9 from *N* = 7 mice) was larger than that in the Con mice (*n* = 15 from *N* = 6 mice, *p* = 0.030). In Con mice, the amplitude of le-IPSCs in the non-ADP cells (*n* = 16 from *N* = 9 mice) was larger than in ADP cells (*n* = 15 from *N* = 6 mice; *p* = 0.0025). *p < 0.05, **p < 0.01. **(D)** Comparison of the I/E ratio of the non-ADP and ADP cells between the Con and FC mice. Comparison of the I/E ratio of the Con and FC mice between the subtypes. The I/E ratio was significantly higher in the FC group (*n* = 15 from *N* = 6 mice) than in the Con group of ADP cells (*n* = 9 from *N* = 7 mice; *p* = 0.020). Between the subtypes, the I/E ratio of the non-ADP cells was significantly larger than that of the ADP cells in Con mice (non-ADP: 0.49 ± 0.17, *n* = 16 from *N* = 9 mice, ADP: 0.047 ± 0.025, *n* = 15 from *N* = 6 mice; Kruskal–Wallis test with Dunn’s *post-hoc* test, *p* = 0.0008). **(E)** The relationship between the le-EPSCs and le-IPSCs amplitude between subtypes in Con mice (open black circles: non-ADP cells, *n* = 15 from *N* = 6 mice; open red triangles: ADP cells, *n* = 9 from *N* = 7 mice). **(F)** The relationship between the le-EPSCs and le-IPSCs amplitude between subtypes in FC mice (filled black circles: non-ADP cells, *n* = 16 from *N* = 9 mice; filled red triangles: ADP cells, *n* = 12 from *N* = 7 mice) *p < 0.05, **p < 0.01, ***p < 0.001. **(G)** Schematic diagram of the aversive learning-induced I/E balance changes. In the Con group, the inhibition input into non-ADP cells was larger than that into ADP cells (left panel). After aversive learning, feedforward inhibition in ADP cells increased, and the I/E balance drastically changed. On the other hand, feedforward inhibition in non-ADP cells slightly decreased, and the I/E balance reversely changed. The I/E balance change may have contributed to the weight of the relative outputs of the non-ADP and ADP cells (right panel).

Aversive learning induces excitation/inhibition balance changes in the excitatory neurons of the prelimbic cortex to the basolateral amygdala (BLA) pathway but not in the infralimbic cortex to the BLA pathway after FC (Arruda-Carvalho and Clem, 2014). However, whether aversive learning regulates E/I balance changes depending on the LA subtype is still unknown. To address this question directly, we investigated the light-evoked feed-forward inhibitory postsynaptic currents via MGN axonal inputs (Figure 5A). To measure the amplitude of le-IPSCs, we performed whole-cell patch-clamp recordings at a holding potential of –40 mV (Figure 5A). We compared the amplitudes of the le-IPSCs between Con and FC mice. The amplitude of le-IPSCs recorded from FC mice was similar to that recorded from Con mice (Con mice: 57.67 ± 10.62 pA, *n* = 24 from *N* = 8 mice, FC mice: 63.44 ± 14.59 pA, *n* = 28 from *N* = 11 mice, Mann–Whitney U test, *p* = 0.79; Figure 5B). Under our experimental conditions with a holding potential of –40 mV, recorded IPSCs may be partially masked because of the large amplitude of EPSCs in some recording cells. To examine whether the feed-forward inhibitory input pattern is different in a target-dependent manner, we first compared the le-IPSCs amplitude between the non-ADP cells and the ADP cells within the Con mice. The le-IPSCs amplitude of the ADP cells was significantly smaller than those of the non-ADP cells within Con mice (ADP: 13.18 ± 9.30 pA, *n* = 9 from *N* = 7 mice, non-ADP: 81.35 ± 12.51 pA, *n* = 15 from *N* = 6 mice, Kruskal–Wallis test with Dunn’s *post-hoc* test, *p* = 0.0025; Figure 5C). On the other hand, the le-IPSCs amplitude of the non-ADP cells was similar to that of the ADP cells within the FC mice (non-ADP: 39.06 ± 10.84 pA, *n* = 16 from *N* = 9 mice; ADP: 88.99 ± 29.61 pA, *n* = 12 from *N* = 7 mice; Kruskal–Wallis test with Dunn’s *post-hoc* test, *p* = 0.43; Figure 5C). This difference could be attributed to a significant increase in inhibitory inputs to ADP cells after aversive learning (Kruskal–Wallis test with Dunn’s *post-hoc* test, *p* = 0.030; Figure 5C). On the other hand, inputs to non-ADP cells after aversive learning tended to decrease (Kruskal–Wallis test with Dunn’s *post-hoc* test, *p* = 0.059; Figure 5C). Based on these observations, we identified the subtype-dependent inhibitory synaptic plasticity after aversive learning. Finally, we evaluated the excitation/inhibition balance changes from the I/E ratio in each excitatory subtype after aversive learning. The I/E ratio was defined as the amplitude of the le-IPSCs divided by the amplitude of the le-EPSCs recorded from the same cells. In the Con mice, the I/E ratio of the non-ADP cells was significantly larger than that of the ADP cells (non-ADP: 0.49 ± 0.17, *n* = 15 from *N* = 6 mice, ADP: 0.047 ± 0.025, *n* = 9 from *N* = 7 mice; Kruskal–Wallis test with Dunn’s *post-hoc* test, *p* = 0.0008; Figure 5D), while in the FC mice, there were no significant differences (non-ADP: 0.20 ± 0.064, *n* = 16 from *N* = 9 mice; ADP: 0.47 ± 0.20, *n* = 12 from *N* = 7 mice; Kruskal–Wallis test with Dunn’s *post-hoc* test, *p* = 0.62; Figure 5D). In the non-ADP cells, the I/E ratio in the FC mice tend to be smaller than that in Con mice (Kruskal–Wallis test with Dunn’s *post-hoc* test, *p* = 0.062; Figure 5D). In the ADP cells the I/E ratio in FC mice was significantly larger than that in Con mice (Kruskal–Wallis test with Dunn’s *post-hoc* test, *p* = 0.020; Figure 5D). To reveal the relationship between le-EPSCs and le-IPSCs, we plotted the amplitude of the le-EPSCs on the horizontal axis and that of the le-IPSCs on the vertical axis. Regarding the relationship between le-EPSCs and le-IPSCs, there was a significant correlation for non-ADP cells, but not for ADP cells (black dashed line: *r* = 0.67, *p* = 0.0068; red line: *r* = 0.18, *p* = 0.65; Figure 5E). In contrast, for the relationship between le-EPSCs and le-IPSCs after FC, there was no correlation between both non-ADP (dashed black line: *r* = 0.23, *p* = 0.39) and ADP (red line: *r* = 0.41, *p* = 0.18; Figure 5F) cells. Taken together, we found that both thalamic excitatory and feed-forward inhibitory inputs differed between the excitatory subtypes. Interestingly, the I/E ratio of the non-ADP cells and the ADP changed reversely after aversive learning. The I/E balance change may have contributed to the weight of the relative outputs of the non-ADP and ADP cells (Figure 5G).

### Disynaptic inhibition is differentially modulated in the two excitatory subtypes

**FIGURE 6:**
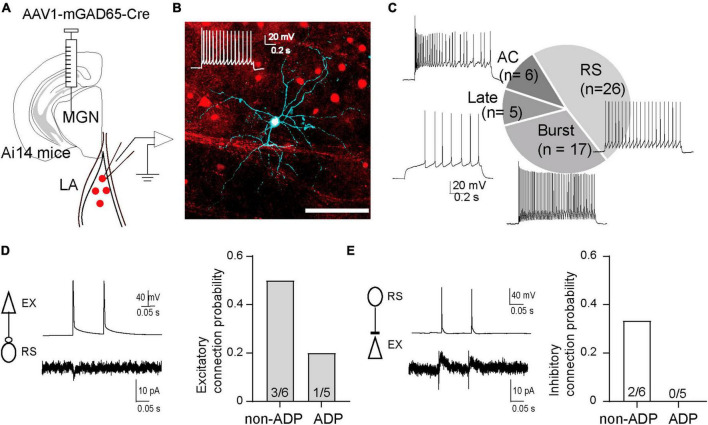
The different inhibitory systems may be involved in the disynaptic inhibition of the two excitatory subtypes. **(A)** Schematic of AAV1-mGAD65-cre injection into the medial geniculate nucleus (MGN) of Ai14 mice. Patch clamp recordings were performed from the labeled cells in the excitatory cells at holding potentials –40 mV. **(B)** Td-tomato was expressed in GABAergic cells. The recorded cells were filled with biocytin and stained by Streptavidin Alexa 647 after fixation. The firing patterns of the recorded cells exhibited Regular spiking (RS). **(C)** The chart showed the number of cells classified as regular spiking (RS: 48.1%), burst spike (Burst: 31.5%), late spike (Late: 9.3%), accommodating type (AC: 11.1%), and representative firing patterns of each type (*N* = 12 from Ai14 mice). **(D)** Averaged EPSCs in the RS cell in response to two successive action potentials of the non-ADP cells (upper panel). Excitatory connection probability from non-ADP, ADP to RS cells. **(E)** Averaged IPSCs in the non-ADP cell in response to two successive action potentials of the RS cells (upper panel). Inhibitory connection probability from RS cells to non-ADP, ADP cells.

The different changes in I/E balance between non-ADP and ADP cells suggest that they receive feed-forward inhibitory inputs from different inhibitory neurons. To address this possibility, we next investigated which types of inhibitory cells are involved in disynaptic inhibition, and how ADP and non-ADP cells form excitatory-inhibitory circuits with inhibitory cells. For that purpose, we used the anterograde transsynaptic AAV system (Zingg et al., 2017). We injected AAV1-mGAD65-Cre (Hoshino et al., 2021) into the MGN of Ai14 mice, and 1–2 weeks after AAV injection, we observed td-tomato labeled cells in the LA (Figures 6A, B). Following this, we performed patch-clamp recordings from the labeled cells and confirmed that many of those recorded cells have the firing patterns of inhibitory cells, as previously reported. To classify the inhibitory cells, we used nine electrophysiological parameters for hierarchical cluster analysis: rmp, threshold, AP amplitude, half-width of AP, AHP, AHP time, Ri, i-ISI, sag ratio (see Materials and Methods, Table 1) although we added some inhibitory cells recorded from wildtype (*n* = 20 from *N* = 14 mice, Supplementary Figure 1). The firing patterns were divided into four types regular spiking (RS: *n* = 26), burst spiking (Burst: *n* = 17), late spike (Late: *n* = 5), and accommodating types (AC: *n* = 6) on the Euclidian linkage distance (Figure 6C). We found that RS cells (*n* = 26) were the prevalent cell type in the disynaptic interneurons. We next investigate the excitatory- inhibitory local circuits involving RS cells and non-ADP or ADP cells, we conducted paired recordings between these cell types. First, we elicited two successive presynaptic spikes in excitatory cells and recorded EPSCs in RS cells. The excitatory connection probability from non-ADP to RS cells was 50% (3 of 6 pairs from *N* = 5 mice), and that from ADP to RS cells was 20% (1 of 5 pairs from *N* = 3 mice; Figure 6D). Second, to examine the inhibitory synaptic connections, we elicited two successive presynaptic spikes in RS cells and compared the IPSCs between in non-ADP and ADP cells. While the inhibitory connection probability for RS to non-ADP cells was 33.3% (2 of 6 pairs from *N* = 5 mice), no connected pair was found for RS to ADP cells (0 of 5 pairs from *N* = 3 mice; Figure 6E). These results suggest that ADP and non-ADP cells form different local circuits from inhibitory cells and that the inhibitory cells involved in the disynaptic inhibition may differ. Taken together, we found that thalamic feed-forward inhibitory inputs differed between the excitatory subtypes.

**TABLE 1:** Comparison of the elctrophysiological properties of the inhibitory cells.

	(a) RS (*n* = 29)	(b) Burst (*n* = 24)	(c) AC (*n* = 12)	(d) Late (*n* = 9)	
Rmp (mv)	−59.67 ± 0.93	−64.80 ± 1.0	−57.04 ± 1.32	−68.47 ± 1.81	b < a**, d < a**** b < c***, d < c****
Threshold (mv)	−35.25 ± 0.70	−42.43 ± 0.61	−37.77 ± 0.82	−39.39 ± 0.87	b < a**, c** d < a**, c***
Half width (ms)	1.15 ± 0.066	0.74 ± 0.047	1.21 ± 0.16	1.67 ± 0.11	b < a**, c*, d****
AP amplitude (mV)	73.80 ± 1.13	84.11 ± 1.17	79.15 ± 1.96	90.26 ± 1.72	a < b****, d**** c < d*
AHP (mV)	−16.71 ± 0.85	−13.09 ± 0.61	−15.33 ± 1.01	−9.75 ± 0.58	b < a*, d < a**** d < c**
AHP time (s)	0.019 ± 0.0030	0.015 ± 0.0028	0.035 ± 0.017	0.019 ± 0.0058	
Sag ratio	0.017 ± 0.0018	0.015 ± 0.0024	0.040 ± 0.0072	0.0047 ± 0.00080	c < a*, d < a** c < b**, d < b* d < c****
i-ISI (s)	0.025 ± 0.0038	0.020 ± 0.0045	0.017 ± 0.037	0.089 ± 0.021	a < d**, b < d*** c < d**
Ri (**Ω**)	213.2 ± 17.59	217.4 ± 26.37	632.5 ± 50.49	134.2 ± 12.41	a < c***, b < c**** d < c****

*p < 0.05,

**p < 0.01,

***p < 0.001,

****p < 0.0001.

## Discussion

The present study uncovered a heterogeneity in the ADP characteristics of excitatory cells within the LA. We classified excitatory cells in the LA into non-ADP and ADP cells based on their ADP amplitude (Figure 1). The two excitatory subtypes showed remarkable electrophysiological differences (Figure 2). After aversive learning, excitatory inputs from the MGN did not increase in both subtypes (Figure 4). However, the feed-forward inhibitory inputs were surprisingly different depending on the subtype, resulting in distinct changes in the I/E balance (Figure 5).

It has been reported that the firing patterns of LA excitatory cells are classified into adaptation and non-adaptation types in rats (Faber et al., 2001; Faber and Sah, 2002). Although most of the non-ADP and ADP cells exhibited non-adaptation firing (Figure 1A), they had significantly different AHP amplitudes (Figure 2G). The difference in AHP size between ADP and no-ADP may be due to the possibility that ADP masks AHP. In addition, the two excitatory subtypes had spines in their dendrites and pyramidal shapes as previously reported (Faber et al., 2001). We found that the non-ADP and ADP cells were similar dendritic morphologies (Figure 3). Although changes in the number and complexity of dendrites may not occur in our experimental time scale, such as just 1 day after FC, morphological plasticity in response to FC would be a critical issue for future study. Notably, in our classification of the excitatory cells, we revealed subtype-dependent differences in electrophysiology and synaptic plasticity following aversive learning.

Lateral amygdala excitatory neurons of rats and mice *in vitro* have previously shown larger postsynaptic excitatory currents in response to MGN-LA pathway stimulation in FC animals than in control animals (McKernan and Shinnick-Gallagher, 1997; Clem and Huganir, 2010; Kim and Cho, 2017; Jeong et al., 2021). However, we did not observe any differences in the le-EPSC between the Con and FC mice (Figure 4F). Under our experimental conditions, we did not include the blocker of inhibitory input in the ACSF, and it is highly likely that the greater inhibitory inputs masked the excitatory inputs. Therefore, the greater inhibition of ADP cells after FC results in a smaller excitatory input, whereas in the control conditions, the greater inhibitory input of non-ADP cells resulted in a smaller excitatory input. These factors may have masked potential differences in EPSCs between the control and FC conditions in our experimental conditions.

Notably, following aversive learning, there was an increase in inhibitory inputs to ADP cells, while the inhibitory inputs to the non-ADP-cells tended to decrease (Figure 5C). Thus, one can speculate that the activity of ADP cells might undergo downregulation, while non-ADP cells might be relatively weighted in terms of output (Figure 5G). This regulation may correspond to the previously reported CSup neurons that are upregulated and CSdown neurons that are downregulated during fear learning (d’Aquin et al., 2022). In their study, the synaptic plasticity of the CSup and Csdown neurons are differentially regulated by somatostatin (SOM) and parvalbumin (PV)-positive interneurons during fear learning.

In the present study, le-IPSCs served as the feed-forward inhibitory input via the MGN synaptic pathway. To elucidate the inhibitory cells contributing to disynaptic inhibition, we investigated AAV1-mGAD65-Cre and identified RS cells as the major cell type involved. It is important to note that this classification was solely based on hierarchical cluster analysis of the electrophysiological parameters; hence, we were unable to align it with the firing patterns of chemically marker-positive interneurons. One of the inhibitory interneuron candidates involved in feed-forward IPSCs is the PV-positive interneurons, which are well-known major interneuron subtypes in the cortex (Kawaguchi and Kubota, 1997; Markram et al., 2004). It has been shown that aversive learning drives synaptic plasticity from PV-positive interneurons to excitatory cells in the LA (Lucas et al., 2016). The other candidates involved in le-IPSCs changes are interneuron-selective interneurons: a vasoactive intestinal peptide (VIP)/or calretinin-positive interneurons. Recent studies have revealed that the largest subtypes of interneurons are not PV-positive interneurons but interneuron-selective interneurons: VIP and/or calretinin-positive interneurons in the LA (Vereczki et al., 2021). In addition, MGN directly innervates VIP-positive interneuron cells in the LA (Krabbe et al., 2019).

Despite having a limited number of paired recordings at our disposal, we studied the local circuits of the RS cells and the two excitatory subtypes. Among the RS-ADP cell pairs, we observed the absence of inhibitory connections from RS to ADP cells in all cases. Conversely, in the RS and non-ADP cells, we found reciprocal connections (Figures 6D, E). This suggests that ADP cells might drive the RS cells and potentially induce disinhibition with respect to non-ADP cells. The distinction in inhibitory cell involvement in disynaptic inhibition may suggest the possibility of alterations in excitatory outputs following aversive learning. It is reported that VIP-positive interneurons play an important role via the disinhibition of excitatory cells during aversive learning in the BLA; that is, VIP-positive interneurons inhibit PV- or SOM-positive interneurons and consequently promote excitatory cell activity during aversive stimulation (Krabbe et al., 2019). The combination of feed-forward inhibition and disinhibition by PV- and VIP-positive interneurons may contribute to the I/E balance in both non-ADP and ADP cells.

In the present study, we investigated excitatory subtype-dependent I/E balance changes via the MGN-LA pathway after aversive learning. During aversive learning, inputs from the thalamus and secondary auditory cortex converge into excitatory cells in the LA and exhibit LTP in *in vitro* experiments (Weisskopf et al., 1999; Humeau et al., 2003). Recent studies have shown that these two inputs play different roles in consolidating long-term fear memory during the early post-learning periods (Lee et al., 2021). However, as there may also be some excitation-inhibition balance differences via the secondary auditory cortex to the LA pathway after aversive learning, it will be important to investigate these differences to reveal the relationship between I/E balance and convergence during aversive learning in the future.

The balance between synaptic excitation and inhibition in neural circuits is regulated by experience-dependent plasticity (Maffei et al., 2004; House et al., 2011; Isaacson and Scanziani, 2011). However, how synaptic plasticity in aversive learning affects the excitation-inhibition balance in the LA remains poorly understood. We found that, following aversive learning, ADP cells exhibited dramatic changes in the I/E balance, whereas non-ADP cells tended to change reversely (Figure 5). This experience-dependent plasticity leads to increased feed-forward inhibitory responses in ADP cells, whereas non-ADP cells could be weighted to the output. Consequently, the information output of non-ADP cells may be tuned to amplify the output of fear-related information (Figure 5G).

In conclusion, using the newly classified excitatory subtypes, we found significant experience-dependent plasticity in a subtype-specific manner, particularly in feed-forward inhibitory responses. Following aversive learning, the I/E balance in ADP cells changed drastically, whereas that in non-ADP cells tended to change in the reverse direction. The shift in I/E balance may contribute to the activation of pathways involved in adaptive behaviors in fear learning. These subtype-dependent changes associated with experience-dependent plasticity may play essential roles in aversive learning.

## Data availability statement

The raw data supporting the conclusions of this article will be made available by the authors, without undue reservation.

## Ethics statement

The animal study was approved by the Institutional Animal Care and Use Committee of Jikei University. The study was conducted in accordance with the local legislation and institutional requirements.

## Author contributions

MM: Conceptualization, Data curation, Formal analysis, Funding acquisition, Investigation, Writing—original draft. SM: Data curation, Formal analysis, Investigation, Writing—review and editing. ST: Data curation, Formal analysis, Funding acquisition, Investigation, Visualization, Writing—review and editing. TN: Data curation, Formal analysis, Funding acquisition, Visualization, Writing—review and editing. AK: Funding acquisition, Resources, Writing—review and editing. HH: Funding acquisition, Resources, Writing—review and editing. AMW: Conceptualization, Funding acquisition, Investigation, Methodology, Project administration, Supervision, Validation, Writing—original draft.
