# Supplementary material for: Excitatory subtypes of the lateral amygdala neurons are differentially involved in regulation of synaptic plasticity and excitation/inhibition balance in aversive learning in mice
Source: Front Cell Neurosci. 2023 Dec 14;17:1292822. doi: 10.3389/fncel.2023.1292822 (PMC10755964; doi:10.3389/fncel.2023.1292822)
Supplement: Supplementary file 1 [file Data_Sheet_1.docx]

Supplementary Material

# Supplementary Figure

## Supplementary Figure


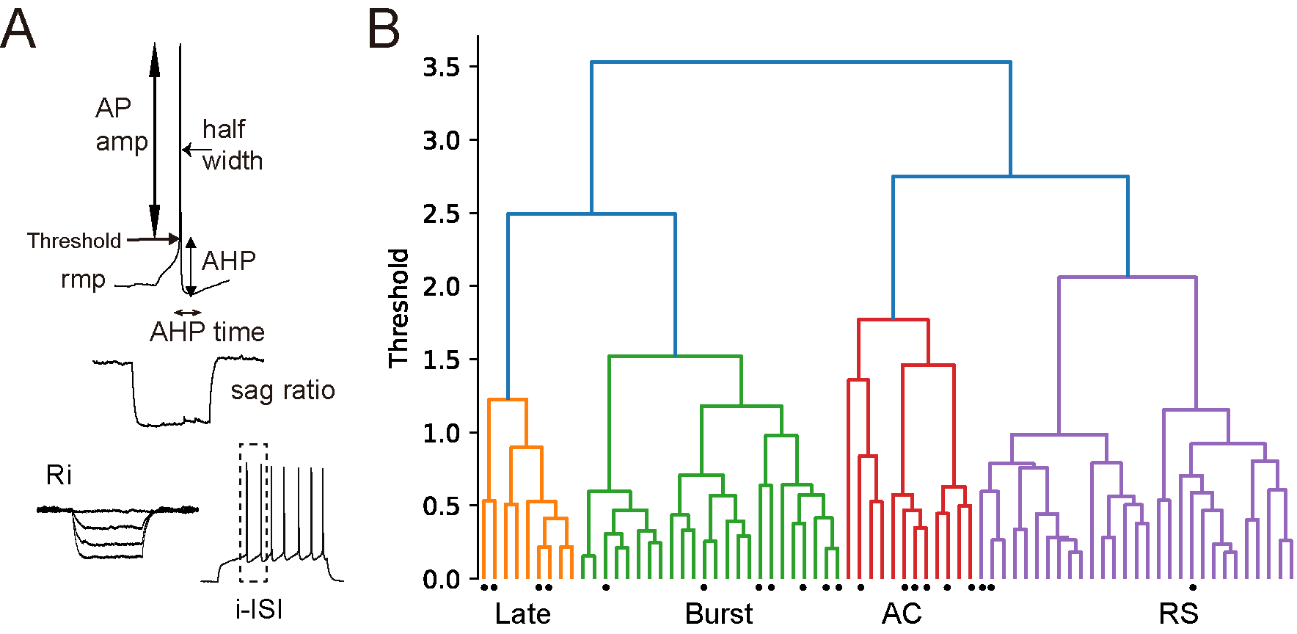


**Supplementary Figure 1.** The hierarchical cluster analysis of the recorded interneurons

A. Nine physiological parameters used for hierarchical cluster analysis were resting membrane potential (rmp), threshold, half-width of action potential (AP), AP amp, after-hyperpolarized potential amplitude (AHP), AHP time, sag ratio, Ri, and i-ISI

B. Interneurons were divided into four groups (orange, green, red, and purple dendrograms) by hierarchical cluster analysis of nine intrinsic properties of the interneurons (labeled cells: n = 54 from N = 12, wild type: black circle, n = 20 from N = 14).
